# Clinical features of suicidal behaviouг in youth with borderline personality disorder

**DOI:** 10.1192/j.eurpsy.2024.625

**Published:** 2024-08-27

**Authors:** V. Kaleda, A. Kuleshov, E. Krylova

**Affiliations:** ^1^Mental Health Research Centre, Moscow, Russian Federation

## Abstract

**Introduction:**

Borderline personality disorder (BPD) in youth has the greatest spectrum of psychopathology and is strictly associated with nonsuicidal self-injury (NSSI) and suicidal behaviour [Guile et al. Adolesc Health Med Ther 2018; 9 199-210; Paris Med. 2019; 55(6):223]. The formation of autoaggressive behaviour and suicidal activity is due to the psychopathological features of BPD, which include affective instability, impulsivity and impaired self-identity.

**Objectives:**

The aim of the study was to investigate the psychopathological features of suicidal behaviour in BPD in youth.

**Methods:**

Clinical and psychopathological examination with assessment of suicidal behaviour at the time of, 6 and 12 months later. For additional psychometric examination of patients we used: SCID-II questionnaires, Barratt Impulsiveness Scale (BIS-11), Toronto Alexithymic Scale (TAS), Columbia Suicide Severity Rating Scale (C-SSRS). Sample: N=62 male and female youth males and females in two equal groups of 31, respectively, with an established diagnosis of BPD and the presence of suicidal behaviour. The mean age of first referral in both groups was 19.1±2.2 years.

**Results:**

This study defined 2 variants of suicidal behaviour in patients with BPD in youth: 1) Expansive - with predominance of impulsiveness (BIS-11 70±3), affective instability, associated with psychosocial factors as a trigger of suicidal activity. Suicidal attempts were made at the height of psychoemotional stress. These patients were characterised by moderately high scores of the C-SSRS scale 2±1, in which patients noted the absence of a plan and specific intentions before the attempt, and a lower incidence of repeated attempts after 6 ((N=6 (19.4%) and 12 months N=10 (32.2%). 2) Rationalistic variant of suicidal behaviour was found in patients with predominance of self-identification disorders, dissociative disorders and high level of alexithymia TAS 81±4.2 in the clinical picture. Suicidal ideation was revealed in all patients, often throughout the entire youth period, and attempts were characterised by thoughtfulness and led to severe consequences, including fatal outcome. Patients with rationalistic variant of suicidal activity had higher C-SSRS scale scores of 4±1, with the presence of suicidal intentions and high frequency of attempt recurrence after 6 (N=11 (35.5%) and 12 months (N=17 (54.8%)).

**Conclusions:**

The variant of suicidal behaviour depended on the degree of severity and correlation of the psychopathological structure of BPD in youth. Less favourable prognosis was characteristic of the rationalistic variant due to the high frequency of repeated attempts. The results obtained require further analysis and contribute to the development of differentiated therapeutic strategies.

**Disclosure of Interest:**

None Declared

